# Lung Cancer Risk in Men and Compliance with the 2018 WCRF/AICR Cancer Prevention Recommendations

**DOI:** 10.3390/nu14204295

**Published:** 2022-10-14

**Authors:** Iwona Hawrysz, Lidia Wadolowska, Malgorzata Anna Slowinska, Anna Czerwinska, Janusz Jacek Golota

**Affiliations:** 1 Department of Human Nutrition, University of Warmia and Mazury in Olsztyn, Sloneczna 45f, 10-718 Olsztyn, Poland; 2 Independent Public Complex of Tuberculosis and Lung Diseases in Olsztyn, 10-357 Olsztyn, Poland; 3 Clinic of Thoracic Surgery, Medical Center Ars Medica, 10-513 Olsztyn, Poland

**Keywords:** lung cancer, prevention, compliance with recommendations, the 2018 WCRF/AICR Score, food-based guidelines, dietary guidelines, case–control study

## Abstract

Lung cancer is the most common and deadly form of cancer worldwide, especially in men. The 2018 World Cancer Research Fund/American Institute for Cancer Research (WCRF/AICR) updated cancer prevention recommendations, and a standard scoring system (2018 WCRF/AICR Score) was published. The purpose of this study was to develop the adapted version of the 2018 WCRF/AICR Score with respect to lung cancer prevention recommendation (Ad-LC WCRF/AICR Score) and to examine the association between lung cancer risk in men and the Ad-LC WCRF/AICR Score as well as its single components. A case–control study was conducted among 439 men aged 45–80 years (187 controls, 252 primary lung cancer cases). Lifestyle and dietary data were collected with a questionnaire including the 62-item food frequency questionnaire (FFQ-6^®^). The Ad-LC WCRF/AICR Score was used as a categorized and continuous variable. Odds ratios (ORs) and 95% confidence intervals (95% CIs) for lung cancer risk were calculated with the partly and fully adjusted model. One component of the score was independently associated with a lower risk of lung cancer in men, regardless of the set of confounders used. In the fully adjusted model following the recommendation “Limit smoking” was associated with a lower risk of lung cancer—in the never smokers by 87% (OR: 0.13; 95% CI: 0.04–0.37; *p* = 0.0002) and in the moderate smokers by 45% (OR: 0.55; 95% CI: 0.33–0.91; *p* = 0.0189) compared with the heavy smokers as a reference. By adding the single components making up the Ad-LC WCRF/AICR Score, the combination of three components or more, reducing the risk of lung cancer compared to lower compliance as a reference by 45% to 78% and by 39% to 66% for intermediate compliance (except two models out of seven) and higher compliance, respectively. In the fully adjusted model, the risk of lung cancer for the total Ad-LC WCRF/AICR Score was lower by 47% (OR: 0.53; 95% CI: 0.32–0.88; *p* = 0.0129) in higher compliance with the score compared to those with the lower compliance. Each one-point increase in the Ad-LC WCRF/AICR Score reduced lung cancer risk by 34% (OR: 0.66; 95% CI: 0.45–0.95; *p* = 0.0267). The results support previous evidence that limiting smoking reduces the risk of lung cancer in men. It also provides an insight into cancer research by showing that following the combined 2018 cancer prevention recommendations related to diet, lifestyle and body fatness was associated with a lower risk of lung cancer in men.

## 1. Introduction

Men usually have a shorter life expectancy compared to women and significantly higher mortality with regard to chronic diseases such as cancers [1]. Lung cancer is the world’s leading cause of cancer death, including in Poland [2,3]. This is largely because it is initially asymptomatic and typically discovered at advanced stages [4]. Both modifiable and non-modifiable risk factors may increase the risk of lung cancer [5,6]. The link between tobacco smoking and lung cancer is by far the strongest and longest-known risk association of a modifiable lifestyle factor with a specific type of cancer [7,8]. Poor diet, occupational exposures, and air pollution may act independently or jointly with tobacco smoking in shaping the descriptive epidemiology of lung cancer [9].

Most of the available studies have focused on the analysis of the relationship between single dietary components and the risk of lung cancer [9,10,11,12,13,14,15,16]. The influence of lifestyle factors (in addition to smoking and occupational exposure in the workplace) [7,17] and the combined impact of diet [18,19,20,21,22,23,24,25,26] on the risk of lung cancer is still limited. An approach that combines lifestyle and dietary factors can be very useful in developing a primary lung cancer prevention strategy.

Given that an estimated 40% of all cancer cases are preventable [27], the World Cancer Research Fund (WCRF) and American Institute for Cancer Research (AICR) published a report in 2018 entitled *Diet, Nutrition, Physical Activity, and Cancer: A Global Perspective* [28]. This report was the basis for the development of the Standardized Scoring System Cancer Prevention Recommendations, which was developed in collaboration with scientists from the US National Cancer Institute (NCI) with members of AICR and WCRF International, and in consultation with the WCRF/AICR Panel Expert and other researchers [7,28]. The proposed 2018 WCRF/AICR Score focuses on eight key lifestyle components, including body weight, physical activity, diet, breastfeeding (optional), and smoking, recommended for some models [7,29]. The WCRF/AICR Continuous Update Project Expert Panel encourage researchers to implement this standardized score in their epidemiologic and clinical studies to enhance the comparability of findings across populations and countries [7,28].

The 2018 WCRF/AICR Score is an a priori approach based on prior knowledge of the health effects of dietary and lifestyle components. To date, there are publications regarding lung cancer risk versus the Mediterranean diet index or healthy eating index [23,30,31,32]. To the authors’ knowledge, no studies have so far been published assessing the relationship between these updated recommendations and lung cancer risk. Furthermore, it is not clear to what extent the total effect of lifestyle factors contributes to the incidence of lung cancer.

Therefore, it seems reasonable to hypothesize that the 2018 WCRF/AICR cancer prevention recommendations may be considered for lung cancer prevention. The purpose of this study was twofold: (i) to develop the adapted version of the 2018 WCRF/AICR Score with respect to lung cancer prevention recommendation (Ad-LC WCRF/AICR Score), and (ii) to examine the association between lung cancer risk in men and the Ad-LC WCRF/AICR Score as well as its single components.

## 2. Materials and Methods

### 2.1. Ethical Statement

This study was approved by the Bioethics Committee of the Faculty of Medical Sciences, University of Warmia and Mazury in Olsztyn on 2 October 2013 (resolution no. 29/2013). All of the subjects gave their written informed consent to participate in the study.

### 2.2. Study Design and Participants

The total case–control sample obtained 439 men (187 lung cancer cases, 252 controls). The inclusion criteria were: (i) men, (ii) age ≥18 years, north-eastern Poland (urban and rural areas) living, (iv) lung tomography or X-ray examination, and (v) consent to participate in the study. The sample ranged in age from 45 to 80 years (mean 62.6), although recruiting was among adult males with no age restrictions. The study participants were enrolled in the study between October 2013 and August 2017. In order to avoid changes in dietary habits or other behaviors, newly diagnosed cases of lung cancer (up to a maximum of 14 days after cancer diagnosis) were included in the study. The sample collection and study design have previously been described in detail [22,23].

### 2.3. Data Collection

All of the lifestyle questions referred to the period 12 months before diagnosis. Some questions, such as about smoking status, referred to a period in the past and present. Since the survey was conducted face-to-face by a trained interviewer, the interview was detailed, and any uncertainties were immediately clarified. The baseline questionnaire gathered data on the following background variables for both cases and controls: current sociodemographic, lifestyles (smoking status, alcohol consumption, physical activity, and occupational exposure in the workplace), family history of lung cancer, and vitamin/mineral supplement use.

#### 2.3.1. Sociodemographic Data

Age, place of residence, education level, and economic status were collected in the initial section of the questionnaire through an individual interview with each participant. The respondents were asked about three factors describing their socioeconomic status (SES). The SES index was calculated as the sum of the values (scores) assigned to the response categories for each SES factor (Table 1). SES index values were logarithmized, and SES tertiles were created to distinguish respondents with low, average, and high SES.

#### 2.3.2. Lifestyle Data

The respondents were asked to describe their physical activity at work and physical activity in their leisure time by choosing one of three categories (Table 2). The self-declared data were then combined, and three categories of overall physical activity were created: low, moderate, and high (Table 3).

To determine smoking status, data on current smoking status, duration, intensity of smoking, and time since cessation were collected. The smoking status was expressed in pack years. The subjects were classified into three groups: never smoker (0 pack years), moderate smoker (2.5–11 pack years), or heavy smoker (>11 pack years). Smoking categories were based on the median value, which was 11 pack years in this sample (at enrollment). Details on calculation pack years were described previously [29] and are given in Supplementary Material.

#### 2.3.3. Body Composition Data

Trained researchers undertook the measurements of body weight (kg) and height (cm), and body mass index (BMI, kg/m^2^) was calculated. Professional equipment and measuring tape were used: for weight—electronic digital scale SECA 799, for measuring height—a portable stadiometer SECA 220, for waist and hip circumference—stretch-resistant tape SECA 201. The method of near-infrared interaction with the FUTREX 6100/XL was used to measure the body fat content (%).

#### 2.3.4. Dietary Data

The 62-item food frequency questionnaire (FFQ-6^®^) in an interviewer-administered version was used [33] to collect data on the frequency of food consumption. The validation procedure of the 62-item FFQ-6^®^ was described by Niedzwiedzka et al. [34]. The respondents were asked about food frequency consumption (6 categories to choose from) within the last 12 months prior to involvement in the study. The frequencies of consumption were recalculated and expressed as times/day as follows: ‘never or almost never’ = 0; ‘once a month or less’ = 0.025; ‘several times a month’ = 0.1; ‘several times a week’ = 0.571; ‘daily’ = 1; ‘several times a day’ = 2 times/day. The frequencies of the consumption of some food items were aggregated, and the following food groups were created: whole grains/vegetables/fruits/beans; fast food/other processed foods high in fat/ starches/sugars; red/processed meats; sugar-sweetened drinks. The sum of the average frequency of food consumption per group (times/day) was then calculated. For these food groups, subjective cut-off points were selected based on tertile distribution within each specific dataset (Table 4). For alcohol consumption, three categories of ethanol consumption were created based on the number of drinks consumed per day: 0 ethanol per day (0 drinks/day), >0–≤28 g of ethanol per day (2 drinks/day), and >28 of ethanol per day (2 drinks/day). All of the cut-offs were made based on the literature-derived components of the proposed 2018 WCRF/AICR Score [7].

### 2.4. Development of the Ad-LC WCRF/AICR Score

The 2018 WCRF/AICR Score described by Shams-White et al. [7] was modified for this analysis, called the Adapted lung cancer WCRF/AICR Score (Ad-LC WCRF/AICR Score). Of the eight recommendations (single components of the score), the current study included seven components with the modification of one component and the addition of one important lung cancer risk factor: (1) normal body weight modified to normal body fat mass, (2) physical activity, (3) eating whole grains/vegetables/fruits/grains, (4) limiting the consumption of fast food/other processed foods high in fat/starches/sugars, (5) limiting red/processed meat consumption, (6) limiting sugar-sweetened beverage consumption, (7) limiting alcohol consumption, and (8) limiting smoking. The breastfeeding component is not relevant to the current study and was not used in the dietary-lifestyle assessment. The indirect measurement of body fat (BMI) was replaced with the direct measurement of body fat using near-infrared interaction (see Section 2.3.3).

When calculating the Ad-LC WCRF/AICR Score, the scoring weights were equally divided among these components. Each recommendation was assigned a score as follows: 1 point for meeting, 0.5 points for partially meeting, and 0 points for not meeting each recommendation. The total scores ranged from 0 to 8 points, with higher scores indicating greater compliance with the Ad-LC WCRF/AICR Score recommendations for lung cancer prevention (Table 4). The single components and their combinations and the total Ad-LC WCRF/AICR score are also presented. The scores were further categorized according to predefined cut-off points [35]: lower compliance: ≤3 points; intermediate compliance: >3–≤5 points; and higher compliance: >5 points.

### 2.5. Statistical Analysis

The adequacy of the sample size related to the current study was checked. The post hoc statistical power was calculated, taking into consideration variables being components of the Ad-LC WCRF/AICR Score. For example, the data were used to compare two groups with means of body fat content (28.0 SD 8.0% vs. 23.0 SD 6.4%), means of frequency of fruit and vegetable consumption (1.1 SD 0.6 times/day vs. 1.7 SD 1.0 times/day), means of frequency of red/processed meat consumption (2.4 SD 1.2 times/day vs. 1.3 SD 1.0 times/day), the occurrence of low physical activity (65.1% vs. 50.4%) and the occurrence of ethanol consumption >28 g/day (28.8% vs. 12.8%). Assuming a 5% significance level, the statistical power was 98%, 95%, 100%, 82% and 54%, respectively. It was therefore concluded that the sample size was appropriate.

The characteristics of the participants were examined using means and standard deviations (SD) for continuous variables and percentages for categorical variables by categories of the Ad-LC WCRF/AICR Score. A chi-squared test was used to evaluate the level of significance of the differences observed in categorical variables and the Kruskal–Wallis test for continuous variables.

The odds ratios (ORs) were used to assess the association between the Ad-LC WCRF/AICR Score as well as its single components and the risk of lung cancer development. The references (OR = 1.00) were the control sample and the bottom categories (lower compliance with the cancer prevention recommendations). The significance level of OR was verified with Wald’s test [36]. The total Ad-LC WCRF/AICR Score was analysed as a categorical variable (for lower, intermediate, and higher compliance) and as a continuous variable (per one point of an increase). Two adjusted models were created for the association between single components, the Ad-LC WCRF/AICR Score and the risk of lung cancer. Model 1 (partly adjusted) included the following as the set of confounders: age (years), BMI (categories: <18.5; 18.5–24.9; 25–29.9; ≥30 kg/m^2^), socioeconomic status (low, average, high), the occurrence of lung cancer in a relative (yes, no, I do not know), occupational exposure in the workplace (yes, no), vitamin/mineral supplement use (no, yes). Model 2 (fully adjusted) included the same confounders as model 1 and single components of the Ad-LC WCRF/AICR Score, excluding the modelled variables as appropriate.

For all tests, the level of statistical significance was considered as *p* < 0.05. Statistical analyses were performed using STATISTICA statistical software (version 13.0 PL; StatSoft Inc., Tulsa, USA; StatSoft, Krakow, Poland).

## 3. Results

The majority of men (254 subjects; 57.9%) had intermediate compliance with the Ad-LC WCRF/AICR Score, while only 39 men (8.9%) had higher compliance. Men with higher compliance with the Ad-LC WCRF/AICR Score compared to men with intermediate or lower compliance were younger, more often lived in urban areas, smoked less, had lower average BMI, waist circumference, and body fat content, as well as a lower frequency of consumption of red/processed meats, sugar-sweetened drinks, fast foods/other processed foods high in fat/starches/sugars and lower ethanol consumption while higher physical activity and frequency of consumption of fruits/vegetables and whole grains/beans (Table 5 and Table 6). In the Supplementary Material frequency of food group consumption by categories of compliance with fruit/vegetable consumption, whole grain/beans consumption, and red/processed meats consumption was shown (Tables S1–S3).

The odds ratios for the single components of the Ad-LC WCRF/AICR Score are shown in Table 7. One component of the score was independently associated with a lower risk of lung cancer in men, regardless of the set of confounders used. In the fully adjusted model (model 2), following the recommendation “Limit smoking” was associated with a lower risk of lung cancer—in the never smokers (scored with 1 point) by 87% (OR: 0.13; 95% CI: 0.04–0.37; *p* = 0.0002) and in the moderate smokers (scored with 0.5 points) by 45% (OR: 0.55; 95% CI: 0.33–0.91; *p* = 0.0189) compared with the heavy smokers as a reference (scored with 0 points).

In the partly adjusted model (model 1), two single recommendations achieved statistical significance (positive or negative). In model 1, following the recommendation “Eat whole grains/vegetables/fruits/beans” was associated with a lower risk of lung cancer in frequent consumers of these foods (scored with 1 point) by 31% (OR: 0.69; 95% CI: 0.53–0.90; *p* = 0.0057) compared to infrequent consumers as a reference (scored with 0 points). In model 1, following the recommendation “Limit red/processed meat intake” was associated with a higher risk of lung cancer—in moderate frequent consumers (scored with 0.5 points) by more than two-fold (OR: 2.04; 95% CI: 1.16–3.58; *p* = 0.0129 and in infrequent consumers (scored with 1 point) by 1.4-fold (OR: 1.40; 95% CI: 1.07–1.97; *p* = 0.0152) in comparison to frequent consumers (scored with 0 points). In the fully adjusted model (model 2) for both recommendations (“Eat whole grains/vegetables/fruits/beans” and “Limit red/processed meat intake”), the statistical significance disappeared after including single components of the Ad-LC WCRF/AICR Score in the set of confounders.

By adding the single components that make up the Ad-LC WCRF/AICR Score, the combination of three components and more, for both intermediate compliance (except model 6 and full model) and higher compliance (no exception), reduced the risk of lung cancer compared to lower compliance as a reference. In the intermediate compliance group, the risk of lung cancer was lower by 45% to 78% (Model 2 OR: 0.55; 95% CI: 0.21–1.00; *p* = 0.0497; Model 3 OR: 0.22; 95% CI: 0.12–0.39; *p* < 0.0001; Model 4 OR: 0.54; 95% CI: 0.32–0.96; *p* = 0.0335; Model 5 OR: 0.49; 95% CI: 0.29–0.82; *p* = 0.0075). In the higher compliance group, the risk of lung cancer was lower by 39% to 66% (Model 2 OR: 0.53; 95% CI: 0.36–0.78; *p* = 0.0013; Model 3 OR: 0.57; 95% CI: 0.32–0.99; *p* < 0.0441; Model 4 OR: 0.40 95% CI: 0.27–0.59; *p* < 0.0001; Model 5 OR: 0.34; 95% CI: 0.17–0.69; *p* = 0.0026; Model 6 OR: 0.61; 95% CI: 0.43–0.85; *p* = 0.0041) (Table 8). In the full model (7), the risk of lung cancer for the total Ad-LC WCRF/AICR Score was lower by 47% (OR: 0.53; 95% CI: 0.32–0.88; *p* = 0.0129) in higher compliance with the score compared to those with lower compliance as a reference. Each one-point increase in the Ad-LC WCRF/AICR Score reduced lung cancer risk by 34% (OR: 0.66; 95% CI: 0.45–0.95; *p* = 0.0267) (Table 8).

## 4. Discussion

In 2018, the World Cancer Research Fund/American Institute for Cancer Research published the Third Expert Report (Diet, Nutrition, Physical Activity, and Cancer: A Global Perspective) [5]. A year later, a standardized scoring system for the association between adherence to these recommendations and cancer prevention was developed and published [7]. The current findings support these recommendations and highlight the possible importance of the interaction of diet-related components and lifestyle factors with lung cancer risk. Regarding the Ad-LC WCRF/AICR Score, in the current study, higher adherence to the score reduced lung cancer risk. This association was found in both approaches—for categories and per one-point increase in the score.

To the best of the authors’ knowledge, this is the first case–control study describing the association between the 2018 WCRF/AICR cancer prevention recommendations and lung cancer risk. The strongest and longest-known modifiable lifestyle risk factor for lung cancer is smoking [7,8,37,38], which was also observed in this study. A protective association was also found between more frequent consumption of whole grains/vegetables/fruits/beans and lung cancer risk in a partially adjusted model. However, this protective association disappeared in the fully adjusted model when other dietary- and lifestyle-related components were included as confounders, including potentially negative components such as red/processed meat [39,40]. Other studies [41,42] have found that the consumption of fruits and vegetables, which are rich in antioxidant vitamins, phenolic compounds, minerals, and fibre, has beneficial effects on respiratory health.

Referring to the single components of the Ad-LC WCRF/AICR Score, one finding must be discussed. In a partially adjusted model, an increased risk of lung cancer in those who followed the “Limit red/processed meat consumption” recommendation was stated. However, this surprising result has an explanation. As shown in the Supplementary Material (Table S3), the lower frequency of red/processed meat consumption (i.e., higher compliance with the recommendations) was accompanied by a significantly lower frequency of consumption of vegetables and fruits. In particular, in the higher compliance with the red/processed meats consumption group, these foods were consumed nearly once a day (on average 0.9 times/day), while vegetables and fruits were consumed slightly above once a day (on average 1.1 times/day). Thus, despite a lower frequency of red/processed meat consumption, a protective effect of vegetables and fruits did not exist [41,42]. Previous studies [4,5,9,32,43,44,45] have shown that the consumption of red and processed meat can increase the risk of lung cancer. In contrast, the European Prospective Investigation into Cancer and Nutrition (EPIC) study [14] found no association between meat or processed meat consumption and lung cancer risk.

Due to the complexity of the diet, the approaches assessing associations with cancer that focus on single dietary habits, foods, or nutrients may not be appropriate because it displays only a part of the association [46,47]. An alternative approach is to focus on the overall dietary patterns that express many different aspects of diet [48] and lifestyle [7,49] combined. Such a holistic approach in the cancer prevention recommendations was used by the WCRF/AICR experts [7,28]. An important point of the WCRF/AICR recommendations was that each recommendation was intended to be one part of a comprehensive package of modifiable lifestyle behaviours that together promote a healthy pattern of diet and physical activity conducive to cancer prevention. Thus, a package of lifestyle behaviours should be considered more important in interpretation and conclusion than single ones. Such holistic conclusions were drawn from studies carried out previously [18,19,20,21,22,23,24,25,26,29,30,31,49]. For example, a study on cardiometabolic health in Polish men showed that a healthy diet combined with an active lifestyle (healthy diet, activity at work, former smoking pattern) was associated with a reduced risk of obesity and metabolic abnormalities despite some unhealthy components, such as the frequent consumption of fried foods [49]. The authors’ previous findings in a cancer-control cohort of men (the same as the current cohort) have shown that health-promoting dietary patterns (Prudent pattern and Mediterranean pattern) may promote a lower risk of lung cancer, even in moderate smokers (smoking 2.5–10 pack years) [29]. The current study casts new light on the association between dietary-lifestyle factors and lung cancer. It showed that a combination of some single dietary-lifestyle factors was associated with reduced lung cancer risk (by approx. a half lower) despite some unhealthy components, for example, higher frequency of red/processed meats consumption, i.e., the beneficial aspects of overall diet and lifestyle prevailed. As in real life, nobody follows all recommendations, and this is good news for lung cancer prevention. A message directed to people can be: “follow as many recommendations as possible”(but it is not necessary to follow all recommendations).

Adherence to a health-promoting lifestyle, including dietary habits, is more common in women than in men [50]. This thesis is supported by the current study, in which only 39 men out of 439 subjects were in the higher adherence score range. Indeed, men seem to have some resistance to following a healthy diet [51]. The current study found that lung cancer risk was lower for total Ad-LC WCRF/AICR Score and demonstrated a protective association between each one-point increase in Ad-LC WCRF/AICR Score and lung cancer risk. To date, the association between adherence to the 2018 WCRF/AICR recommendations and lung cancer incidence has not been assessed, but it has already been examined for the 2007 WCRF/AICR recommendations. A 2007 report: Food, nutrition, physical activity, and the prevention of cancer: a global perspective, presented eight general and two specific recommendations for cancer prevention [52]. Since then, several studies have evaluated the association between adherence to these recommendations and lung cancer risk. In several studies, adherence to the 2007 recommendations for diet, physical activity, and weight control was associated with reduced lung cancer risk [53,54,55]. However, a standardized scoring method to determine adherence to the 2007 WCRF/AICR recommendations had not been previously developed, so each study developed its own version of the scoring. To increase the comparability of results across populations and countries, a standardized scoring system for WCRF/AICR cancer prevention recommendations was developed in 2018. The current study is the first (and so far, the only) study to evaluate the association between adherence to the 2018 WCRF/AICR recommendations and lung cancer risk in men. To date, there have been few studies evaluating the association between updated recommendations and the risk of other cancers. Studies on total cancer incidence in Sweden [56] and cancers of the colon in Spain [57] and the USA [58], breast in Spain [59] and the USA [60], and prostate in Spain [61], similarly to the current study, found a positive association with adherence to the 2018 WCRF/AICR recommendations. In contrast, one arm of the EPIC project focused on breast cancer in Europe [35], and a clinical-control study focused on chronic lymphocytic leukaemia in Spain [62] found no association with adherence to the 2018 WCRF/AICR recommendations.

### Strengths and Limitations

The current study may have some potential limitations that should be noted. This is a case–control design, and the results may be subject to information bias. Lifestyle data, such as dietary and physical activity, were collected through a questionnaire, and the results may also be susceptible to recall bias [62]. Moreover, the use of a food frequency questionnaire is limited by the use of a fixed food list. Furthermore, different interpretations of questions regarding food items and the frequency of consumption by individuals can also be taken into consideration [63,64]. To overcome these limitations, trained interviewers helped participants to answer more accurately while completing the questionnaire. Although the method is not free from measurement error, previous studies using repeated and mixed-method dietary assessment confirmed the utility of food frequency questionnaires in exploring associations between diet and chronic disease risk [64,65]. The use of a simple scoring index (Ad-LC WCRF/AICR Score) should also be considered a potential limitation of the study. The score was based on the sum of several risk factors in the authors’ own adaptation or whose association with lung cancer risk is not proven. Such an approach may have weakened the association of scores in the study. Smoking was included as a proven risk factor for lung cancer in the scoring to strengthen the interpretation of the score. Moreover, the indirect measure of body fat (BMI) was replaced with a direct measure of body fat using near-infrared interaction. BMI does not fully account for body composition, such as differences in muscle proportion and different types of fatness in patients with the same BMIs, especially in chronically ill patients [66]. Men who are diagnosed with lung cancer may already exhibit preclinical weight loss.

The current study also has several strengths. It is the first study of its kind in Central Europe to describe the relationship between WCRF/AICR recommendations and lung cancer risk in men. Cases and controls were selected according to the inclusion criteria for the study population described previously [29]; cases were recruited at diagnosis and before treatment. We used the validated FFQ-6^®^ food frequency questionnaire. This type of questionnaire does not provide information on estimated portion size but represents typical eating habits of Poles [22,23,24,67,68,69]. In Poland, there has only been one validated semi-quantitative and comprehensive FFQ (165-item FFQ^®^) [70], but it causes a heavy burden on the respondent (an interview takes 3–4 h). Conducting the 165-item FFQ^®^ among men with lung cancer would have been too difficult. The available data on potential cancer risk factors allowed the survey results to be adjusted for confounders, but unmeasured or residual confounders cannot be ignored.

## 5. Conclusions

The current study provides insight into cancer research by showing that following the combined 2018 cancer prevention recommendations related to diet, lifestyle, and body fatness was associated with a lower risk of lung cancer in men. It also provides new support for previous evidence that limiting smoking reduces the risk of lung cancer in men. The study found that a combination of some single dietary-lifestyle factors was associated with reduced lung cancer risk despite some unhealthy components. Based on these findings in developing pro-healthy strategies, the message directed to the public can be: “follow as many recommendations as possible to prevent lung cancer”.

## Figures and Tables

**Table 1:** Description of the socioeconomic status factors scoring—values assigned to the response categories.

Socioeconomic Factors	Categories	Scoring
Place of residence	rural	1
	sub-urban	2
	urban	3
Education level	primary	1
	secondary	2
	higher	3
Economic situation	below average	1
	average	2
	above average	3

**Table 2:** Description of the categories of physical activity at work and in leisure time.

Physical Activity	Categories	Description
at work	low	more than 70% of working time spent sedentary or retired
	moderate	50% of working time spent sedentary and 50% of working time spent in an active manner
	high	70% of working time spent in an active manner or physical work related to great exertion
at leisure time	low	sedentary for most of the time, watching TV, reading books, walking 1–2 h/week
	moderate	walking, bike riding, gymnastics, gardening, light physical activity performed 2–3 h/week
	high	bike riding, jogging, gardening, and sports activities involving physical exertion performed more than 3 h weekly

**Table 3:** Estimate the overall physical activity after combining data based on self-reported physical activity at work and physical activity in leisure time.

		Physical Activity at Work		
		Low	Moderate	High
Physical activity in leisure time	Low	low	low	moderate
	Moderate	low	moderate	moderate
	High	moderate	moderate	high

**Table 4:** Scoring of the official 2018 WCRF/AICR Score and its adapted version with respect to lung cancer (Ad-LC WCRF/AICR Score).

2018 WCRF/AICR Recommendations	Operationalization of Recommendations	Scoring of the 2018 WCRF/AICR Score (Points)	Ad-LC WCRF/AICR Recommendations	Adapted Operationalization of the Recommendations	Scoring of the Ad-LC WCRF/AICR Score (Points)
1. Be a healthy weight	**BMI (kg/m^2^):**		1. Have a healthy body fat	**Body fat content (%):**	
	18.5–24.9	0.5		11.0–20.0	1
	25–29.9	0.25		20.1–24.9	0.5
	<18.5 or ≥30	0		<11.0 or >25.0	0
	**Waist circumference (cm (in)):**				
	Men: <94 (<37); Women: <80 (<31.5)	0.5			
	Men: 94–<102 (37–<40); Women: 80–<88 (31.5–<35)	0.25			
	Men: ≥102 (≥40); Women: ≥88 (≥35)	0			
2. Be physically active	**Total moderate-vigorous physical activity (min/wk):**		2. Be physically active	**Overall physical activity:**	
	≥150	1		High	1
	75–<150	0.5		Moderate	0.5
	<75	0		Low	0
3. Eat a diet rich in whole grains, vegetables, fruit, and beans	**Fruits and vegetables (g/day):**		3. Eat whole grains/vegetables/fruits/beans	**Fruits and vegetables ^1^ (times/day ^#^):**	
	≥400	0.5		Tertile 3 (≥1.435)	0.5
	200–<400	0.25		Tertile 2 (>0.921 < 1.435)	0.25
	<200	0		Tertile 1 (≤0.921)	0
	**Total fibre (g/day):**			**Whole grains and beans ^2^ (times/day ^#^)^:^**	
	≥30	0.5		Tertile 3 (≥1.050)	0.5
	15–<30	0.25		Tertile 2 (>0.200 < 1.050)	0.25
	<15	0		Tertile 1 (≤0.200)	0
4. Limit consumption of “fast foods” and other processed foods high in fat, starches, or sugars	**Percent of total kcal from ultra-processed foods:**		4. Limit consumption of fast foods/other processed foods high in fat/starches/sugars	**“Fast foods” and other processed foods high in fat, starches and sugars ^3^ (times/day ^#^):**	
	Tertile 1	1		Tertile 1 (≤2.421)	1
	Tertile 2	0.5		Tertile 2(>2.421 < 4.038)	0.5
	Tertile 3	0		Tertile 3 (≥ 4.038)	0
5. Limit consumption of red and processed meat	**Total red meat (g/wk) and processed meat (g/wk):**		5. Limit consumption of red/processed meats	**Red meat and processed meats ^4^ (times/day ^#^):**	
	Red meat <500 and processed meat <21	1		Tertile 1 (≤1.392)	1
	Red meat <500 and processed meat 21–<100	0.5		Tertile 2 (>1.392 < 2.359)	0.5
	Red meat >500 or processed meat ≥100	0		Tertile 3 (≥2.359)	0
6. Limit consumption of sugar-sweetened drinks	**Total sugar-sweetened drinks (g/day):**		6. Limit consumption of sugar-sweetened drinks	**Sugar-sweetened drinks ^5^ (times/day ^#^):**	
	0	1		Tertile 1 (≤0.000)	1
	>0–≤250	0.5		Tertile 2 (>0.000 ≤ 0.025)	0.5
	>250	0		Tertile 3 (>0.025)	0
7. Limit alcohol consumption	**Total ethanol (g/day):**		7. Limit alcohol consumption	**Ethanol (g/day):**	
	0	1		0 (0 drinks/day)	1
	>0–≤28 (2 drinks) males and ≤14 (1 drink) females	0.5		>0–≤28 (2 drinks/day) males	0.5
	>28 (2 drinks) males and >14 (1 drink) females	0		>28 (>2 drinks/day) males	0
8. (Optional) For mothers: breastfeed your baby, if you can	**Exclusively breastfed over a lifetime for a total of:**		8. (Optional) For mothers: breastfeed your baby, if you can	NA	NA
	6+ months	1			
	>0–<6 months	0.5			
	Never	0			
9. Limit smoking	NI	NI	9. Limit smoking	**Smoking pack years:**	
				Never (0 pack years)	1
				Moderate smoker (>0–11 pack years)	0.5
				Heavy smoker (>11 pack years)	0
**Total Score Range 0–8**			**Total Score Range 0–8**		

Food components included into food groups to calculate Ad-LC WCRF/AICR Score: ^1^ All kinds of fruits and all kinds of vegetables (potatoes not included); ^2^ Wholemeal wheat or rye bread, seeded loaves, pumpernickel, wholemeal cracker bread, buckwheat groats, barley, brown rice, wholemeal pasta, etc., and beans;; ^3^ Fast food, fried food, margarine for baking, frying, spreading; mayonnaise and salad dressings; white bread, white bread rolls, semolina, milled barley, white rice and pasta, rice flakes, etc.; chocolate, chocolate sweets and chocolate bars, boiled sweets, hard caramels, jellied sweets, fudge, biscuits, cream cakes, fruit cakes, sponge cakes, cheesecakes, doughnuts, poppy-seed cakes, muffins, croissants, ice-creams and custard, etc.; ^4^ Pork, beef, veal, wild boar, venison, quail, mallard, hare, etc.; ^5^ Sweetened beverages and energy drinks; ^#^ The consumption frequency of food groups (categories) was expressed as times/day (values) as follows: ‘never or almost never’ = 0; ‘once a month or less’ = 0.025; ‘several times a month’ = 0.1; ‘several times a week’ = 0.571; ‘daily’ = 1; ‘several times a day’ = 2 times/day; NI—Not included in the official 2018 WCRF/AICR Recommendations; NA—Not Applicable to men.

**Table 5:** Baseline characteristics of the cancer-control sample by categories of compliance with the Ad-LC WCRF/AICR Score (%).

Variable	Total (0–8 Points)	Compliance with the Ad-LC WCRF/AICR Score			
		Lower (≤3 Points)	Intermediate (>3–≤5 Points)	Higher (>5 Points)	*p*-Value
Sample size	439	146	254	39	
Sample percentage	100	33.2	57.9	8.9	
Age (years), mean (SD)	62.6 (7.2)	62.8 (7.3)	62.9 (7.0)	60.3 (8.1)	<0.0001
Place of residence					0.0258
rural	34.6	43.8	30.7	25.6	
sub-urban	46.0	41.1	49.2	43.6	
urban	19.4	15.1	20.1	30.8	
Education level					0.1018
primary	23.7	30.8	20.5	18.0	
secondary	58.8	54.1	61.8	56.4	
higher	17.5	15.1	17.7	25.6	
Economic situation					
below average	20.7	23.3	20.5	12.8	0.3331
average	63.3	65.1	61.8	66.7	
above average	16.0	11.6	17.7	20.5	
Socioeconomic status (SES index) ^a^					0.1008
low	53.5	60.3	51.6	41.0	
average	19.8	16.4	22.0	18.0	
high	26.7	23.3	26.4	41.0	
Family history of lung cancer among relatives					0.0925
yes	20.7	21.2	18.5	33.3	
no	73.8	713.3	77.6	59.0	
I do not know	5.5	7.5	3.9	7.7	
Occupational exposure in the workplace					0.8394
yes	31.2	29.5	32.3	30.8	
no	68.8	70.5	67.7	69.2	
Vitamin/mineral supplements use					0.3731
yes	10.7	8.2	11.4	15.4	
no	89.3	91.8	88.6	84.6	
BMI (kg/m^2^), mean (SD)	27.7 (7.2)	28.5 (5.2)	27.4 (4.8)	26.0 (3.4)	<0.0001
<18.5	2.3	1.4	3.1	0.0	0.0272
18.5–24.9	28.5	27.4	27.2	41.0	
25.0–29.9	36.4	30.1	38.9	43.6	
≥30.0	32.8	41.1	30.7	15.4	
Waist circumference (cm), mean (SD)	100.6 (12.8)	102.8 (7.6)	100.3 (12.6)	94.1 (10.1)	<0.0001
<94	29.6	23.9	29.9	48.7	0.0014
94–<102	20.5	28.9	20.5	30.8	
≥102	49.9	38.8	49.6	20.5	

SD—standard deviation; ^a^ SES index was calculated based on place of residence, educational level and declared economic situation (details are given in Section 2.3.1); BMI—Body Mass Index; *p*-Value—level of significance verified with a chi^2^ test (categorical variables) or a Kruskal–Wallis’ test (continuous variable).

**Table 6:** Components of the Ad-LC WCRF/AICR Score by categories of compliance with this score in the cancer-control sample: means (SDs) and sample distribution (%).

Variable	Total (0–8 Points)	Compliance with the Ad-LC WCRF/AICR Score			
		Lower (≤3 Points)	Intermediate (3–≤5 Points)	Higher (>5 Points)	*p*-Value
Sample size	439	146	254	39	
Body fat content (%)	26.6 (7.9)	28.0 (8.0)	26.3 (7.8)	23.0 (6.4)	<0.0001
11.0–20.0	18.4	10.3	19.7	41.0	<0.0001
20.1–24.9	60.4	71.9	59.1	25.6	
<11.0 or >25.0	21.2	17.8	21.3	33.4	
Overall physical activity					
low	51.7	65.1	50.4	10.3	<0.0001
moderate	37.6	32.2	37.8	56.4	
high	10.7	2.7	11.8	33.3	
Smoking in pack years	12.4 (7.4)	14.5 (7.3)	12.0 (7.0)	7.3 (6.7)	<0.0001
0 (never smoker)	12.5	8.2	11.4	35.9	<0.0001
>0–11 (moderate smoker)	57.4	49.3	62.2	56.4	
>11 (heavy smoker)	30.1	42.5	26.4	7.7	
Fruits and vegetables (times/day)	1.3 (0.7)	1.1 (0.6)	1.3 (0.7)	1.7 (1.0)	<0.0001
Tertile 1	34.4	42.5	32.3	17.9	0.0065
Tertile 2	32.6	34.2	31.5	33.3	
Tertile 3	33.0	23.3	36.2	48.7	
Whole grains and beans (times/day)	0.8 (0.8)	0.5 (0.7)	0.9 (0.8)	1.2 (0.9)	0.1070
Tertile 1	35.8	54.1	28.7	12.8	<0.0001
Tertile 2	31.0	28.8	32.3	30.8	
Tertile 3	33.3	17.1	39.0	56.4	
Fast foods/other processed foods high in fat/starches/sugars (times/day)	3.3 (1.7)	4.4 (1.6)	3.3 (1.5)	1.7 (1.0)	<0.0001
Tertile 1	33.6	11.6	37.8	76.9	<0.0001
Tertile 2	33.5	23.3	40.9	23.1	
Tertile 3	33.9	65.1	21.3	0	
Red/processed meats (times/day)	2.0 (1.2)	2.4 (1.2)	1.8 (1.1)	1.3 (1.0)	<0.0001
Tertile 1	34.6	16.4	39.8	69.2	<0.0001
Tertile 2	32.6	33.6	33.9	20.5	
Tertile 3	32.8	50.0	26.4	10.3	
Sugar-sweetened drinks (times/day)	0.1 (0.3)	0.2 (0.4)	0.1 (0.2)	0.1 (0.2)	<0.0001
Tertile 1	50.8	24.7	61.8	76.9	<0.0001
Tertile 2	20.0	25.3	18.9	7.7	
Tertile 3	29.2	50.0	19.3	15.4	
Ethanol (g/day)					
0	23.0	15.7	23.6	46.2	<0.0001
>0–≤28	59.5	55.5	64.6	41.0	
>28	17.5	28.8	11.8	12.8	

SD—standard deviation; Frequencies expressed as times/day are presented after recoding dietary data as follows: ‘never or almost never’ = 0; ‘once a month or less’ = 0.025; ‘several times a month’ = 0.1; ‘several times a week’ = 0.571; ‘daily’ = 1; ‘several times a day’ = 2 times/day; *p*-Value—level of statistical significance verified with a chi^2^ test (categorical variables) or a Kruskal–Wallis’ test (continuous variables).

**Table 7:** Odds ratios (95% confidence intervals) of lung cancer occurrence for single components of Ad-LC WCRF/AICR Score.

Component Included in the Model	Categories	Scores	Cancer-Control Sample (*n* = 439)			
			Model 1	*p*-Value	Model 2	*p*-Value
‘Have a healthy body fat’	11.0–20.0% of body fat	0	Ref.		Ref.	
	20.1–24.9% of body fat	0.5	0.69 (0.36; 1.31)	0.2581	0.71 (0.36; 1.41)	0.3263
	<11.0 or >25.0% of body fat	1	0.86 (0.59; 1.28)	0.4609	0.83 (0.55; 1.25)	0.3705
‘Be physically active’	Low	0	Ref.		Ref.	
	Moderate	0.5	0.70 (0.44; 1.13)	0.1432	0.88 (0.53; 1.47)	0.6237
	High	1	0.73 (0.49; 1.08)	0.1110	0.82 (0.54; 1.24)	0.3445
‘Limit smoking’	Heavy smoker	0	Ref.		Ref.	
	Moderate smoker	0.5	0.53 (0.33; 0.87)	0.0116	0.55 (0.33; 0.91)	0.0189
	Never smoker	1	0.14 (0.05; 0.39)	0.0001	0.13 (0.04; 0.37)	0.0002
‘Eat whole grains/ vegetables/fruits/beans’	Infrequent consumption	0	Ref.		Ref.	
	Moderate frequency consumption	0.5	0.76 (0.44; 1.35)	0.3547	0.90 (0.50; 1.63)	0.7301
	Frequent consumption	1	0.69 (0.53; 0.90)	0.0057	0.77 (0.58; 1.03)	0.0810
‘Limit consumption of fast foods/other processed foods high in fat/starches/sugars’	Frequent consumption	0	Ref.		Ref.	
	Moderate frequency consumption	0.5	1.07 (0.63; 1.81)	0.8014	1.08 (0.60; 1.91)	0.8150
	Infrequent consumption	1	0.90 (0.69; 1.19)	0.4830	0.93 (0.68; 1.27)	0.6389
‘Limit consumption of red/processed meats’	Frequent consumption	0	Ref.		Ref.	
	Moderate frequency consumption	0.5	2.04 (1.16; 3.58)	0.0013	1.78 (0.98; 3.23)	0.0575
	Infrequent consumption	1	1.40 (1.07; 1.97)	0.0152	1.18 (0.87; 1.62)	0.2830
‘Limit consumption of sugar-sweetened drinks’	Frequent consumption	0	Ref.		Ref.	
	Moderate frequency consumption	0.5	0.80 (0.41; 1.57)	0.0512	0.56 (1.25; 0.57)	0.5659
	Infrequent consumption	1	1.10 (0.85; 1.43)	0.4503	0.99 (0.78; 1.26)	0.9514
‘Limit alcohol consumption’	Frequent consumption	0	Ref.		Ref.	
	Moderate frequency consumption	0.5	0.92 (0.50; 1.70)	0.8013	0.89 (0.45; 1.74)	0.8874
	Infrequent consumption	1	1.13 (0.76; 1.67)	0.5440	0.99 (0.63; 1.53)	0.9477

Model 1 was adjusted for: age (years), BMI (categories: <18.5; 18.5–24.9; 25–29.9; ≥30 kg/m^2^), socioeconomic status (low, average, high), the occurrence of lung cancer among a relative (yes, no, I do not know), occupational exposure in the workplace (yes, no); vitamin/mineral supplement use (yes, no); Model 2 = Model 1 + an adjustment for single components of the Ad-LC WCRF/AICR Score excluding modelled variable as appropriate; *p*-Value—the level of statistical significance verified with Wald’s test.

**Table 8:** Odds ratios (95% confidence intervals) of lung cancer occurrence for the combination of components of Ad-LC WCRF/AICR Score.

Components Included in the Model	Scores	Cancer-Control Sample (*n* = 439)	
		Adjusted Model	*p*-Value
Model 1: ‘Have a healthy body fat’ + ‘Be physically active’	≤0.5	Ref.	
	>0.5–≤1	1.04 (0.57; 1.88)	0.9036
	>1	0.69 (0.47; 1.02)	0.0634
Model 2: Model 1 + ‘Limit smoking’	≤1	Ref.	
	>1–<2	0.55 (0.21; 1.00)	0.0497
	≥2	0.53 (0.36; 0.78)	0.0013
Model 3: Model 2 + ‘Eat whole grains/vegetables/fruits/beans’	<2	Ref.	
	≥2–<3	0.22 (0.12; 0.39)	<0.0001
	≥3	0.57 (0.32; 0.99)	0.0441
Model 4: Model 3 + ‘Limit fast food/other processed foods high in fat/starches/sugars’	≤3	Ref.	
	>2–<3	0.54 (0.32; 0.96)	0.0335
	≥3	0.40 (0.27; 0.59)	<0.0001
Model 5: Model 4 + ‘Limit consumption of red/processed meats’	<3	Ref.	
	>3–≤4	0.49 (0.29; 0.82)	0.0075
	>4	0.34 (0.17; 0.69)	0.0026
Model 6: Model 5 + ‘Limit consumption of sugar-sweetened drinks’	≤3	Ref.	
	>3–≤4	0.93 (0.57; 1.53)	0.0708
	>4	0.61 (0.43; 0.85)	0.0041
Model 7 (full): Model 6 + ‘Limit alcohol consumption’	≤3	Ref.	
	>3–≤5	0.87 (0.54; 1.38)	0.5873
	>5	0.53 (0.32; 0.88)	0.0129
1-point increase		0.66 (0.45; 0.95)	0.0267

Model adjusted for: age (years), BMI (categories: <18.5; 18.5–24.9; 25–29.9; ≥30 kg/m^2^), socioeconomic status (low, average, high), the occurrence of lung cancer among a relative (yes, no, I do not know), occupational exposure in the workplace (yes, no); vitamin/mineral supplement use (yes, no); *p*-Value—the level of statistical significance verified with Wald’s test.

## Data Availability

Not applicable.

## Supplementary Materials

The following supporting information can be downloaded at: https://www.mdpi.com/article/10.3390/nu14204295/s1, Table S1. Frequency of food group consumption (times/day; mean (SD)) by categories of compliance with fruit/vegetable consumption^#^ in the case-control sample; Table S2. Frequency of food group consumption (times/day; mean (SD)) by categories of compliance with whole grain/bean consumption^#^ in the case-control sample; Table S3. Frequency of food group consumption (times/day; mean (SD)) by categories of compliance with red/processed meat consumption^#^ in the case-control sample [71].
