# Supplementary material for: Lung Cancer Risk in Men and Compliance with the 2018 WCRF/AICR Cancer Prevention Recommendations
Source: Nutrients. 2022 Oct 14;14(20):4295. doi: 10.3390/nu14204295 (PMC9607029; doi:10.3390/nu14204295)
Supplement: Supplementary file 1 [file nutrients-14-04295-s001.zip › nutrients-1923785-supplementary.pdf]

Lung Cancer Risk in Men and Compliance with the 2018 WCRF/AICR Cancer Prevention Recommendations

by Iwona Hawrysz, Lidia Wadolowska, Malgorzata Anna Slowinska, Anna Czerwinska and Janusz Jacek Golota

Supplementary material

Pack-years of smoking calculation

Data regarding smoking status was collected for all subjects, including current smoking status, duration, the intensity of smoking, and time since cessation. The pack-years of smoking were calculated by multiplying the average number of packs of cigarettes smoked per day (intensity) by the number of years the person had smoked [71]. Smoking status pack-years were classified into three groups: never smoker (0 pack-years), moderate smoker (2.5–11 pack-years) or heavy smoker (> 11 pack-years). These categories were based on the median value, which, in this sample, was equal to 11 pack-years (at enrolment).

Example of calculation:

A man reported smoking 30 cigarettes a day for ten years.
The calculated number of pack-years is: 10 × 30/20 = 15 pack-years
10—years of smoking
30—number of cigarettes per day
20—number of cigarettes in one pack

Table S1. Frequency of food group consumption (times/day; mean (SD)) by categories of compliance with fruit/vegetable consumption # in the case-control sample

Variable	Total (0-8 points)	Compliance with fruit/vegetable consumption			
		Lower (0 points)	Intermediate (0.25 points)	Higher (0.5 point)	p-Value
Sample size	439	151	143	145	
Fruits and vegetables	1.3 (0.7)	0.6 (0.2)	1.2 (0.2)	2.0 (0.6)	<0.0001
**Other dietary components of the Ad-LC WCRF/AICR Score**					
Whole grains and beans	0.8 (0.8)	0.5 (0.6)	0.7 (0.6)	1.2 (1.0)	<0.0001
Fast foods/other processed foods high in fat/starches/sugars	3.3 (1.7)	3.1 (1.6)	3.5 (1.8)	3.5 (1.8)	0.0866
Red/processed meats	2.0 (1.2)	1.6 (1.0)	2.4 (1.3)	2.0 (1.3)	<0.0001
Sugar-sweetened drinks	0.1 (0.3)	0.1 (0.2)	0.1 (0.3)	0.1 (0.3)	0.9530

SD—standard deviation; # To determine compliance with the Ad-LC WCRF/AICR Score, the frequency of fruit/vegetable consumption (times/day) was divided according to tertile distribution: tertile 1 (≤0.921), tertile 2 (>0.921 <1.435), tertile 3 (≥1.435).

Table S2. Frequency of food group consumption (times/day; mean (SD)) by categories of compliance with whole grain/bean consumption # in the case-control sample

Variable	Total (0-8 points)	Compliance with whole grain/bean consumption			
		Lower (0 points)	Intermediate (0.25 points)	Higher (0.5 point)	*p*-Value
Sample size	439	157	139	143	
Whole grains and beans	0.8 (0.8)	0.1 (0.1)	0.6 (0.2)	1.7 (0.7)	<0.0001
**Other dietary components of the Ad-LC WCRF/AICR Score**					
Fruits and vegetables	1.3 (0.7)	0.9 (0.5)	1.3 (0.6)	1.6 (0.8)	<0.0001
Fast foods/other processed foods high in fat/starches/sugars	3.3 (1.7)	3.8 (1.6)	3.4 (1.8)	2.7 (1.6)	<0.0001
Red/processed meats	2.0 (1.2)	1.9 (1.0)	1.9 (1.1)	2.1 (1.0)	0.6680
Sugar-sweetened drinks	0.1 (0.3)	0.2 (0.3)	0.1 (0.3)	0.1 (0.3)	0.6965

SD—standard deviation; # To determine compliance with the Ad-LC WCRF/AICR Score, frequency of whole grain/bean consumption (times/day) was divided according to tertile distribution: tertile 1 (≤2.000), tertile 2 (>0.200<1.050, tertile 3 (≥1.050).

Table S3. Frequency of food group consumption (times/day; mean (SD)) by categories of compliance with red/processed meat consumption # in the case-control sample

Variable	Total (0-8 points)	Compliance with red/processed meat consumption			
		Lower (0 points)	Intermediate (0.5 points)	Higher (1 point)	*p*-Value
Sample size	439	144	143	152	
Red/processed meats	2.0 (1.2)	3.3 (1.0)	1.8 (0.3)	0.9 (0.4)	<0.0001
**Other dietary components of the Ad-LC WCRF/AICR Score**					
Fruits and vegetables	1.3 (0.7)	1.5 (0.7)	1.2 (0.6)	1.1 (0.7)	<0.0001
Whole grains and beans	0.8 (0.8)	0.8 (0.8)	0.8 (0.8)	0.8 (0.8)	0.9106
Fast foods/other processed foods high in fat/starches/sugars	3.3 (1.7)	3.8 (1.8)	3.5 (1.6)	2.7 (0.8)	<0.0001
Sugar-sweetened drinks	0.1 (0.3)	0.2 (0.4)	0.1 (0.2)	0.1 (0.3)	0.0618

SD—standard deviation; # To determine compliance with the Ad-LC WCRF/AICR Score, frequency of red meat/processed meat consumption (times/day) was divided according to tertile distribution: tertile 1 (≤1.392), tertile 2 (>1.392 <2.359), tertile 3 (≥2.359).
